# Brain Network to Placebo and Nocebo Responses in Acute Experimental Lower Back Pain: A Multivariate Granger Causality Analysis of fMRI Data

**DOI:** 10.3389/fnbeh.2021.696577

**Published:** 2021-09-09

**Authors:** Yu Shi, Shaoye Cui, Yanyan Zeng, Shimin Huang, Guiyuan Cai, Jianming Yang, Wen Wu

**Affiliations:** ^1^Department of Rehabilitation, Zhujiang Hospital, Southern Medical University, Guangzhou, China; ^2^Department of Intensive Care Unit, Zhujiang Hospital, Southern Medical University, Guangzhou, China; ^3^Department of Radiology, Zhujiang Hospital, Southern Medical University, Guangzhou, China

**Keywords:** placebo analgesia, nocebo hyperalgesia, GCA, reward system, dopamine, anxiety, opioid

## Abstract

**Background and Objective**: Placebo and nocebo responses are widely observed. Herein, we investigated the nocebo hyperalgesia and placebo analgesia responses in brain network in acute lower back pain (ALBP) model using multivariate Granger causality analysis (GCA). This approach analyses functional magnetic resonance imaging (fMRI) data for lagged-temporal correlation between different brain areas.

**Method**: After completing the ALBP model, 20 healthy subjects were given two interventions, once during a placebo intervention and once during a nocebo intervention, pseudo-randomly ordered. fMRI scans were performed synchronously during each intervention, and visual analog scale (VAS) scores were collected at the end of each intervention. The fMRI data were then analyzed using multivariate GCA.

**Results**: Our results found statistically significant differences in VAS scores from baseline (pain status) for both placebo and nocebo interventions, as well as between placebo and nocebo interventions. In placebo network, we found a negative lagged-temporal correlation between multiple brain areas, including the dorsolateral prefrontal cortex (DLPFC), secondary somatosensory cortex area, anterior cingulate cortex (ACC), and insular cortex (IC); and a positive lagged-temporal correlation between multiple brain areas, including IC, thalamus, ACC, as well as the supplementary motor area (SMA). In the nocebo network, we also found a positive lagged-temporal correlation between multiple brain areas, including the primary somatosensory cortex area, caudate, DLPFC and SMA.

**Conclusion**: The results of this study suggest that both pain-related network and reward system are involved in placebo and nocebo responses. The placebo response mainly works by activating the reward system and inhibiting pain-related network, while the nocebo response is the opposite. Placebo network also involves the activation of opioid-mediated analgesia system (OMAS) and emotion pathway, while nocebo network involves the deactivation of emotional control. At the same time, through the construction of the GC network, we verified our hypothesis that nocebo and placebo networks share part of the same brain regions, but the two networks also have their own unique structural features.

## Introduction

Placebo and nocebo phenomena are commonly reported (Drici et al., 1995; Colloca et al., 2013). Nocebo and placebo responses are defined as negative and positive behavioral, emotional, and cognitive modulation of outcomes (Colloca and Grillon, 2014). These phenomena occur frequently in research and clinical practice (Ernst, 2016). and they are best studied for their effects on pain (Jensen et al., 2012). While placebo response is widely understood to have analgesic effects, nocebo response is thought to be hyperalgesic effect (Benedetti et al., 2003). Both have been found to have therapeutic effects when used in combination or singly (Lesser et al., 2004; Kam-Hansen et al., 2014).

The development of brain functional imaging technology has greatly improved research in psychology and neuroscience (Shi et al., 2019). Brain imaging technology is now widely used to study placebo analgesia and nocebo hyperalgesia (Atlas et al., 2012; Amanzio et al., 2013; Freeman et al., 2015; Wagner et al., 2020). It has been proposed that placebo response enhances activation of the thalamus (THS), insular cortex (IC), amygdala (AMYG), anterior cingulate cortex (ACC), and brainstem (Peciña and Zubieta, 2015; Peciña et al., 2015). A meta-analysis of 25 studies on placebo response found that placebo analgesia suppresses activity in some brain areas, consisting of the dorsal anterior cingulate cortex (dACC), IC, THS, AMYG, striatum, as well as the lateral prefrontal cortex (PFC). Additionally, the activation of some brain areas, including the dorsolateral prefrontal cortex (DLPFC), left IC, rostral anterior cingulate cortex (rACC), ventromedial prefrontal cortex (VMPFC), periaqueductal gray (PAG), and the striatum, correlates with placebo response (Atlas and Wager, 2014). Abnormal hippocampus (HP) activation has been associated with nocebo effects. The HP is involved in anxiety release. During significant increases in anxiety release, the HP elevates pain signals, triggering stress responses (Bingel et al., 2011). Although some brain areas overlap in the above research conclusions, it is still difficult to reach a consistent conclusion, which needs to be further investigated.

Studies have found numerous cross-links between placebo and nocebo effects. Thus, to better understand how nocebo hyperalgesia and placebo analgesia effects are influenced by brain network mechanisms, it is necessary to study the relationship between these phenomena (Freeman et al., 2015). To date, few investigations have made direct comparisons between nocebo and placebo responses in the same individual (Bingel et al., 2011; Benedetti et al., 2014; Kong and Benedetti, 2014). Some studies indicate that the placebo and nocebo responses have separate brain networks. Bingel et al. (2011) opined that the HP is remarkably activated by nocebo response but not by placebo response (Colloca and Benedetti, 2016). However, a study by Scott et al. (2008) opined that the placebo analgesia could remarkably activate brain areas closely related to opioid-mediated analgesia system (OMAS) and the dopamine system, whereas the nocebo hyperalgesia remarkably deactivates these areas. van de Sand et al. opined that nocebo responses are associated with the rolandic operculum, IC, and periaqueductal gray (PAG; van de Sand et al., 2018). Current literature suggests great differences in placebo and nocebo networks, but some brain regions are involved in both, including IC and PAG. Due to variations in experimental designs, brain networks induced by the same phenomenon vary across studies and need more careful analyses. Based on previous studies, we hypothesized that the placebo network and nocebo network should share some of the same brain areas, but the two networks should also have their own unique structural features.

In-depth study of brain mechanisms requires exploration of direct connectivity (Hamilton et al., 2011; Shi et al., 2019). Granger causality analysis (GCA; Zhao et al., 2016), a resourceful approach for abstracting connectivity from time series data, has been widely used to assess dependencies and interrelationships between networks. This method leverages on estimated linear regression modeling and assumes that if past information on X predicts the future on Y, and is more accurate than considering past information on Y alone, then X is a “Granger cause” of Y (Granger, 1969), GCA provides the possibility to study directional associations between diverse brain regions, and can dynamically observe brain network changes (Hamilton et al., 2011; Liao et al., 2011; Zhao et al., 2016; Shi et al., 2019). Deshpande et al. (2009) used multivariable GCA (mGCA) to analyze time-series fMRI data, and assess the dynamics of functional neural networks. While this method is now widely used (Deshpande et al., 2008; Liao et al., 2011; Shi et al., 2019), it has not been applied in placebo analgesia or nocebo hyperalgesia mechanisms.

The ACC is a pivotal structure of the brain that participates in higher brain network functions. In pain-linked networks, the ACC primarily participates in emotional motivation along with pain cognitive attention. Some ACC sections might also function in identifying pain perception components (Wager et al., 2016). The ACC is extensively linked to IC, as well as primary somatosensory cortex area (S1), and therefore processes pain-associated signals originating in IC (Craig et al., 2000; Price, 2000). Moreover, the ACC has an extensive range of nerve fiber connections with key brain parts aligned to the limbic system, comprising the IC, HP, and AMYG (Shi et al., 2019). The ACC is additionally involved in the reward, as well as dopamine systems, and also influences placebo response (Koban et al., 2013). A positive correlation between ACC activation and placebo effect intensity has been reported, indicating ACC’s critical role in placebo brain networks (Peciña et al., 2015). Therefore, studying the ACC may uncover the key nodes in brain network regarding the modulation of nocebo hyperalgesia and placebo analgesia responses.

Here, we used an acute lower back pain (ALBP) model (Shi et al., 2015) to explore the mechanisms involved in nocebo hyperalgesia and placebo analgesia responses in the brain. Findings from this study will improve our comprehension of the brain networks that modulate nocebo hyperalgesia and placebo analgesia responses.

## Materials and Methods

### Participants

Subjects were enrolled *via* adverts in the Zhujiang Hospital. The subjects resided in one place (southern region, Guangzhou, Guangdong Province, China). All participators were right-handed. The criteria of inclusion constituted: (1) participator had not been part of any prior psychological investigation; (2) participator body mass index should be in normal weight (18.5–23.9); (3) participator had not experienced any medical or psychiatric condition, consisting of depression along with mania in the prior 4 weeks; (4) participator did not have pain, consisting of dysmenorrhea, or not have been under medication, consisting of antipyretics or sleeping pills in the prior 4 weeks; (5) participator should not have been on medications that affect the central nervous function at the nerve or vascular levels in the previous 4 weeks; and (6) participator scored less than 50 on the self-rating anxiety scales (SAS) and the self-rating depression scales (SDS; a score of less than 50 reflects “candidate mentally normal”). Participators were not enrolled when they had: (1) organic brain disease; (2) craniocerebral damage history; (3) dependence on drugs; (4) aggressive neurological condition; (5) metal component in body; (6) claustrophobia; or (7) had used pain killers in the prior 4 weeks. Ethical approval for this work was granted by the ethics committee of Zhujiang Hospital affiliated to Southern Medical University, China (World Medical Association, 2013). All subjects granted written informed consent prior to taking part in the study. Participants were allowed to withdraw their data from the investigation in case of concerns about the inherent deception needed in the experimental design. No subjects opted out.

### Sample Size Estimation

According to previous literature, reliable conclusions can be obtained when the sample size is about 24 (Desmond and Glover, 2002). In this study, we set the sample size included in the final analysis to 20.

### Experimental Procedures

Two patches for providing psychological suggestions were designed. One was labeled “analgesic patch” (positive expectation), and the other “algetic patch” (for negative expectation). The two patches resembled the analgesic patch often adopted in clinical practice.

**Figure 1:**
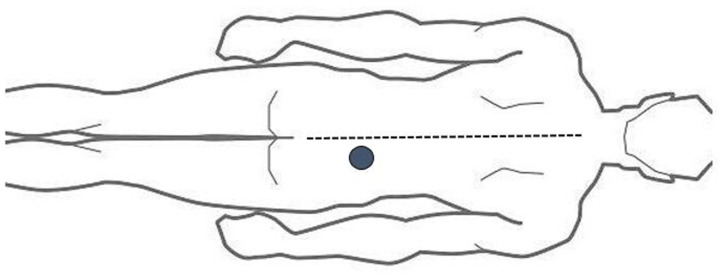
Acute lower back pain (ALBP) model location.

The ALBP model is based on our previous investigation (Shi et al., 2015) and involved location of an injection point 2 cm lateral to the spinous process (SP) of the fourth lumbar vertebra. Next, filling of an in-dwelling 24-G needle was done with 10 ml of 5% sterile hypertonic saline, followed by connection to a computer-modulated power injector (Spectris Solaris EP; Medrad, Inc., United States) using a long connecting tube. It was then vertically inserted 1.5 cm deep into the located injection point. Following the elapse of 1 min, intramuscular administration of the hypertonic saline into the ALBP subject was done with a computer-modulated power injector. The injection consisted of a bolus injection of 0.1 ml in 5 s, followed by a continuous injection at 0.15 ml/min to produce persistent ALBP (Shi et al., 2015; see Figure 1).

#### Training Session

We familiarized the subjects with ALBP, as well as VAS (visual analog scales) for self pain rating. The extent of pain was scored on a 10-cm VAS anchored scale with 0 reflecting “no pain” whereas 10 reflected “worst pain imaginable” (10). Pain unpleasantness was assessed on a 10-cm in-house mood-anchored scale, where 0 designated “infinitely small” while 10 designated “excruciating” (10). Additionally, participant discomfort was also monitored to avoid adverse reactions. At the end of the experiment, behavioral tests were performed where subjects reported pain score changes due to the interventions.

#### Behavioral Conditioning Session

We informed every subject about the aim of the investigation, i.e., to explore the analgesic influences of the analgesic patch along with the algetic influences on the algetic group on their pain encounters. To better acclimatize subjects to the experimental settings, the process was done in the MRI room. Subjects were informed that one of the two patches would be applied to the right foot during ALBP. The subjects could then begin experiencing pain changes on the basis of the patch, and the order of the patch was to reduce the order effect.

After induction of stable ALBP, subjects were required to focus on screen captions. Whenever subjects had an analgesic patch, we displayed the “Please experience the effect of the analgesic patch” statement on the screen: When they had an algetic patch, the following statement would be shown on the screen: “Please experience the effect of the algetic patch.” After the stimulation, VAS would be shown on the screen for subjects’ self-report pain scores. In reality, we lowered the speed of hypertonic saline injection for subjects with analgesic patches and increased it for those with algetic patches. We selected 20 subjects who differentiated the pre-intervention from the post-intervention of the algetic influences from algetic patches or the analgesic influences from analgesic patches to proceed with the downstream investigation.

#### Scanning Session

We informed the subjects that the procedures of the scan session were similar to the prior session, procedures (behavioral conditioning session). However, in reality, we designed the scan session to test placebo and nocebo responses evoked by expectations induced in the behavioral conditioning session. Apart from hypertonic saline adjustment, all other procedures were the same as in the previous session. After establishing ALBP, the impacts of placebo or nocebo were triggered, followed by the collection of MRI data. Brain anatomical scans were collected prior to fMRI scans. To this end, initial (normal) fMRI scans of the subjects were done for 6 min. Afterward, ALBP was stimulated in each subject’s right lower back muscle and after stabilization, an fMRI scan was done to explore the subject’s status of pain. Next, two fMRI scans for every ALBP subject were conducted, one during analgesic patch induction and the other during algetic patch stimulation pseudo-randomly. ALBP was continuously maintained during scanning. To maximize the impacts of the prior intervention, a 10-min time interval was kept between the two scans. The primary output of this study consisted of VAS along with fMRI signal changes resulting from placebo or nocebo influences.

**Figure 2:**
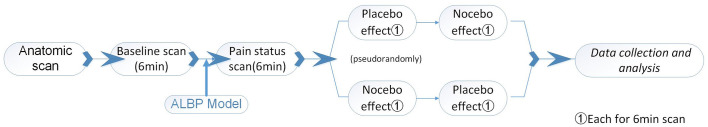
The experimental paradigm (Scanning Session) for the subjects.

In the scanning, subjects with analgesic patches were required to focus on the “Please experience the effect of the analgesic patch, the scanning process is 6 min” statement on the screen. Subjects with algetic patches were shown the statement: “Please experience the effect of the algetic patch, the scanning process is 6 min.” Following each stimulus, VAS was displayed on screen for pain self-scoring (Figure 2).

### Brain Imaging

Brain imaging was done at the Radiology Department of Zhujiang Hospital, Southern Medical University, China. The structural along with the functional scans were assessed with a 3.0 T Philips Achieva MRI system (Royal Philips Electronics) equipped with an 8-channel head array coil for echo planar imaging. Acquired images were axial, as well as parallel to the bicommissural line, covering the entire brain. Images of the structure were acquired before functional scanning with a T1-weighted fast spin echo sequence at a repetition time/echo time of 25/3 ms, flip angle of 30°, Matrix of 256 × 256, thickness of 5 mm, slice of 24, slice gap of 0.7 mm. Oxygenation level of blood-dependent functional images were acquired with a T2*-weighted, single shot, gradient recalled echo planar imaging sequence at a repetition time/echo time of 2,000/35 ms, flip angle of 90°, Matrix of 64 × 64, thickness of 5 mm, slice of 24, slice gap of 0.7 mm, NSA of 1,180 time points for an overall of 360 s).

### Preprocessing of Functional MRI Data

Preprocessing of the fMRI image data along with analysis was done on MATLAB R2013bh with the data processing assistant of resting-state fMRI (DPARSF)^1^. Preprocessing steps of the Blood oxygen level-dependent (BOLD) time series consisted of removing the first 10 volumes, correction of slice-time, correction of motion, normalizing to templates of Montreal Neurological Institute (MNI), averaging data points with their neighbors, removing the linear trend and finally filtering of the temporal band-pass. We discarded the first 10 volumes of every real-scan to get rid of the non-equilibrium impacts of magnetization, as well as allow subjects to adjust to the scanning setting. We adopted courses of motion time to select subjects’ head movements with <2 mm translation along with 2° rotation, which we employed in the downstream analyses. For each participant, normalization of functional images was done with the templates of symmetric echo planar imaging and resampling done at a 3 mm × 3 mm × 3 mm resolution. Spatial smoothing of the normalized functional images was done at a 6 mm full width at half maximum (FWHM) Gaussian kernel. Lastly, removing of voxel-wise linear trend along with pass filtering of the temporal band (0.01–0.08 Hz) were done to minimize very-low-frequency drift, as well as high-frequency noise effects (Shi et al., 2015, 2019).

### Definition of Seed Region

The selection of data on the ACC for the ROI (3 × 3 × 3 mm^3^) was used as previously described (Margulies et al., 2007). Selection of the MNI brain area coordinates was done as the central voxel ROI (*x* = −5, *y* = 25, *z* = −10).

### GCA Process

Here, we adopted Granger causality to evaluate directed connectivity between the seed regions’ reference time series and every voxel’s time series within the entire brain. Bivariate GCA along with multivariate GCA were done with the REST-GCA in the REST toolbox (Shi et al., 2019)^2^.

### Bivariate GCA

Bivariate linear autoregressive model of two time-variant processes, *X* and *Y* was considered:

$$Y_{t}=\sum_{i\;=\;1}^{p}A_{i}X_{\left(t-i\right)}+\sum_{i=1}^{p}B_{i}Y_{\left(t-i\right)}+CZ_{t}+\varepsilon_{t}$$

$$X_{t}=\sum_{i\;=\;1}^{p}A_{i}^{,}Y_{\left(t-i\right)}+\sum_{i=1}^{p}B_{i}^{,}X_{\left(t-i\right)}+C^{/}Z_{t}+\varepsilon_{t}^{'}$$

Based on Granger definitions (Granger, 1969), one time-variant process, *X*, “Granger causes” another process of time-variant, *Y*, designates the across up to *p* temporal lags; if past information on the *X* predicts the future of *Y* with better accuracy than can be achieved from past information on *Y* itself (Deshpande et al., 2009; Shi et al., 2019). *A*_i_ and $A_{\text{i}}^{\prime }$ constitute coefficients of signed-path. *B*_i_ and $B_{\text{i}}^{\prime }$ designate coefficients of autoregression. ε_t_ and $\varepsilon_{\text{i}}^{\prime }$ constitute residuals. *Z*_t_ designates covariates, consisting of head motion, time series along with global trends from specific brain regions. The time series *X*_t_ remarkably Granger results in the time series *Y*_t_ if the signed-path coefficient *A*_i_ is remarkably larger or smaller than zero. On the contrary, *Y*_t_ might be defined as a significant Granger cause to *X*_t_ when the coefficient of signed-path is remarkably larger or smaller relative to zero (Shi et al., 2019). This GCA approach was used to determine brain parts whose time courses estimate the ensuing activity of ACC and those whose activity is forecasted *via* the preceding activity of ACC during the intervention stimulus (Shi et al., 2019). The time series of the seed region in the model consisted of preprocessed data of fMRI that was abstracted from a sphere’s center on the peak of the ACC ROI (3 mm diameter, centered at −5, 25, −10). The time-directed estimation between the BOLD time series across a lag of one TR (2,000 ms), was estimated to optimize the temporal resolution of neural influence estimates. Lastly, comparisons of voxel-wise of the consequent coefficients of GCA fit signed-path across groups (placebo/nocebo vs. baseline pain and placebo vs. nocebo) were done with the *t*-tests across the entire imaging volume (Hamilton et al., 2011; Shi et al., 2019; see Figure 4).

**Figure 3:**
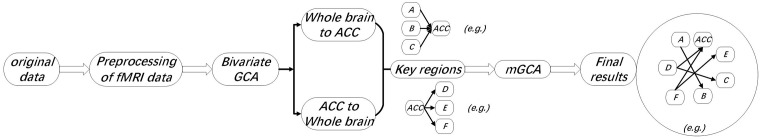
Flow chart of functional magnetic resonance imaging (fMRI) data analysis.

**Figure 4:**
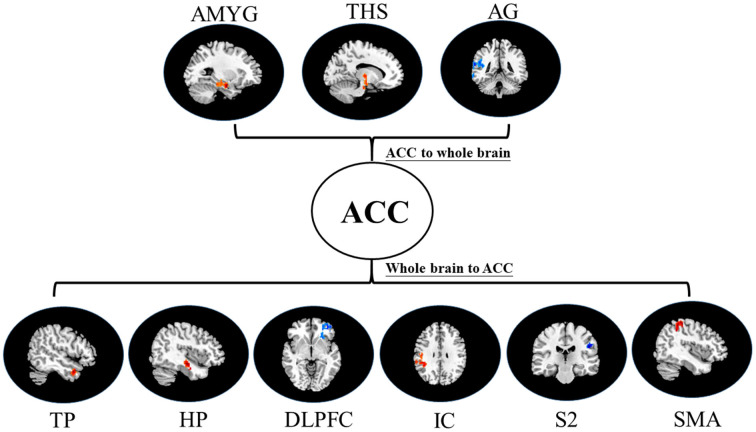
Map of bivariate GCA brain in placebo vs. pain of GCA fit signed-path coefficients. AMYG, amygdala; THS, thalamus; AG, angular gyrus; TP, temporal pole; HP, hippocampus; DLPFC, dorsolateral prefrontal cortex; IC, insular cortex; S2, secondary somatosensory cortex area; SMA, supplementary motor area.

### Multivariate GCA

The definition of bivariate GCA given above can be extended to multivariate settings through the generic multivariate autoregressive model shown:

$$Y_{lt}=\sum_{i=1}^{p}A_{11}^{i}Y_{1}(t-i)+...+\sum_{i=1}^{p}A_{1n}^{i}Y_{n}(t-i)+C_{1}Z_{t}+\varepsilon_{t}$$

$$Y_{nt}=\sum_{i=1}^{p}A_{n1}^{i}Y_{1}(t-i)+...+\sum_{i=1}^{p}A_{nn}^{i}Y_{n}(t-i)+C_{n}Z_{t}+\varepsilon_{t}$$

This approach was used to analyze the brain areas’ REST-GCA voxel time courses (those exhibiting lagging or leading temporal correlations with the activity of ACC in the intervention stimulus (Shi et al., 2019). For every subject, we abstracted preprocessed data of fMRI from peak voxel locations of brain parts that exhibited differential temporal relationships with the ACC across different groups. The data of time-series for every subject were then subjected to multivariate GCA. The consequent coefficients of GCA signed-path typified the strength along with the direction of temporal relationships among the structures (Hamilton et al., 2011; Shi et al., 2019; see Figure 3).

### Statistical Analysis

SPSS statistical suite was used for descriptive statistical analysis (mean ± SD). Voxel-wise assessment (ACC to entire brain and entire brain to ACC) differences across different groups were computed with the two-tailed, paired *t*-tests (*p* ≤ 0.05) and were corrected for multiple comparisons [false discovery rate (FDR), *p* ≤ 0.05]. GCA signed-path coefficient differences across different groups were calculated with the two-tailed, paired *t*-tests (*p* ≤ 0.05), the results also need to be remarkably expressed at the subgroup level. *p* ≤ 0.05 signified statistical significance, and were two tailed, paired tests.

## Results

Twenty healthy subjects (12 male), aged 20–33 participated in the study. We found a statistically significant difference in VAS scores between pain status and placebo response (*p* ≤ 0.001, Table 1). We also found a statistically remarkable difference in VAS scores between pain status and nocebo response (*p* ≤ 0.001, Table 1). At the same time, we also found that the VAS scores of placebo response and nocebo response were also statistically different (*p* ≤ 0.001, Table 1).

**Table 1:** Summary of data statistics of the 20 subjects.

Characteristic		*T*-value
*n*	20	
Age	24.70 ± 2.77	
Gender (female/male)	8/12	
Pain status VAS	4.10 ± 1.25	
Placebo VAS	2.50 ± 1.28	
Nocebo VAS	5.50 ± 1.35	
*T*-test VAS Pain VS Placebo	*P* < 0.001	12.726
*T*-test VAS Pain VS Nocebo	*P* < 0.001	9.756
*T*-test VAS Placebo VS Nocebo	*P* < 0.001	19.504

### Bivariate GCA

**Figure 5:**
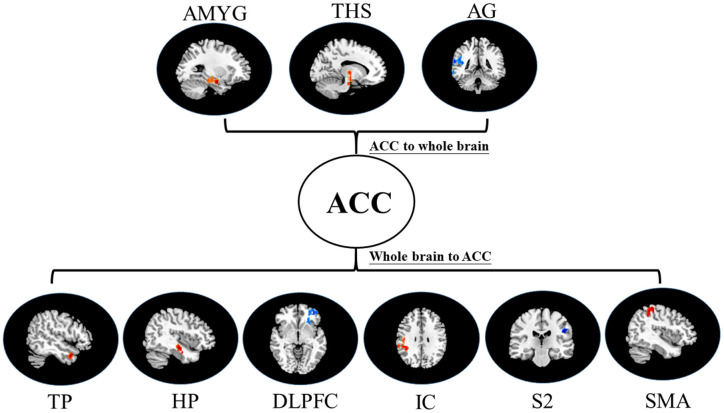
Map of bivariate GCA brain in nocebo relative to pain of GCA fit signed-path coefficients. THS, thalamus; TP, temporal pole; HP, hippocampus; DLPFC, dorsolateral prefrontal cortex; IC, insular cortex; SMA, supplementary motor area; CAU, caudate; S1, primary somatosensory cortex area.

**Figure 6:**
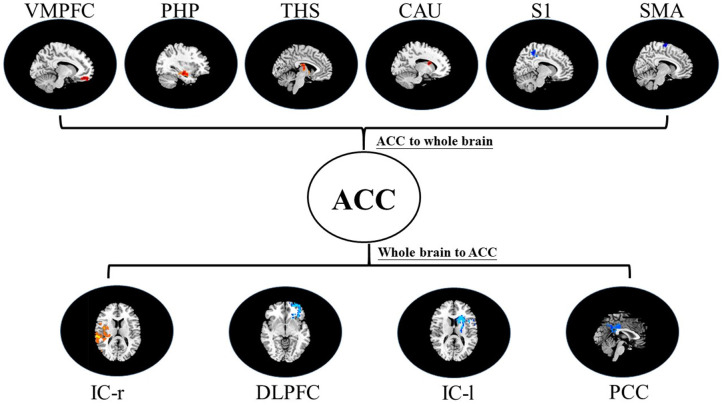
Map of bivariate GCA brain in placebo relative to nocebo of GCA fit signed-path coefficients. VMPFC, ventromedial prefrontal cortex; PHP, parahippocampal gyrus; THS, thalamus; CAU, caudate; S1, primary somatosensory cortex area; SMA, supplementary motor area; IC, insular cortex; DLPFC, dorsolateral prefrontal cortex; PCC, posterior cingulate cortex.

i. In placebo conditions, bivariate ACC to entire brain GCA illustrated that ACC activity forecasted subsequently increased activation in the AMYG and THS. This assessment also revealed that ACC activity predicts subsequently decreased activation in the angular gyrus (AG). After that, the entire brain to ACC GCA exhibited that activity in the temporal pole (TP), HP, IC, and supplementary motor area (SMA), forecasted ensuing elevations in ACC activity to a remarkably greater extent in placebo relative to pain. This assessment also indicated that increased activity in the DLPFC and secondary somatosensory cortex area (S2) predict the ensuing decrease in ACC activity in placebo (Table 2, Figure 4).
ii. In the nocebo condition, bivariate ACC to entire brain GCA depicted that ACC activity forecasted subsequently increased activation in the THS and DLPFC. This assessment additionally depicted that ACC activity predicts ensuing activation decrease in TP and caudate (CAU). After that, the entire brain to ACC GCA illustrated that HP, S1, and SMA activity forecasted the ensuing increase in ACC activity to a remarkably greater extent in the nocebo relative to pain. This analysis found that escalating activity in IC and DLPFC predicts the ensuing decrease in ACC activity in nocebo (Table 3, Figure 5).
iii. In differences between placebo and nocebo conditions, bivariate ACC to the entire brain GCA depicted that ACC activity forecasted subsequently increased activation in the VMPFC, parahippocampal gyrus (PHP), THS, and CAU. This assessment additionally depicted that ACC activity predicts the ensuing activation decrease in S1 and SMA. After that, entire brain to ACC GCA illustrated that right IC activity forecasted the ensuing increase in ACC activity to a remarkably greater extent in the placebo relative to nocebo. This analysis found that escalating activity in left IC, DLPFC, and posterior cingulate cortex (PCC) predict the ensuing decrease in ACC activity (Table 4, Figure 6).

**Table 2:** Data of bivariate GCA of placebo vs. pain of brain GCA of fit signed-path coefficients.

		MNI				
BA	R/L	*X*	*Y*	*Z*	Voxel	Z-score
ACC to whole brain						
AMYG	L	−27	−9	−21	163	6.0855
THS	L	−12	−15	3	131	4.8428
AG	R	63	−45	18	142	−6.9961
Whole brain to ACC						
TP	R	54	6	−30	136	5.8062
HP	R	42	−15	−15	825	7.3076
DLPFC	L	−39	51	0	297	−6.7557
IC	R	45	−39	18	661	6.8854
S2	L	−54	−24	30	139	−6.3224
SMA	R	42	−27	60	295	6.0544

*BA, Brodmann areas; MNI, Montreal Neurological Institute. AMYG, amygdala; THS, thalamus; AG, angular gyrus; TP, temporal pole; HP, hippocampus; DLPFC, dorsolateral prefrontal cortex; IC, insular cortex; S2, secondary somatosensory cortex area; SMA, supplementary motor area*.

**Table 3:** Data of bivariate GCA of nocebo vs. pain of brain GCA of fit signed-path coefficients.

		MNI				
BA	R/L	*X*	*Y*	*Z*	Voxel	Z-score
ACC to whole brain						
TP	R	57	0	−39	151	−5.6085
THS	L	−27	−24	−3	199	5.4073
CAU	R	3	9	15	246	−5.722
DLPFC	L	−30	33	12	238	4.7791
Whole brain to ACC						
IC	L	−36	0	−6	130	−4.3511
HP	R	42	−15	−3	106	5.332
DLPFC	R	51	51	−3	241	−5.1932
S1	L	−66	−9	24	137	7.519
SMA	R	12	24	45	226	6.683

*BA, Brodmann areas; MNI, Montreal Neurological Institute. TP, temporal pole; THS, thalamus; CAU, caudate; DLPFC, dorsolateral prefrontal cortex; IC, insular cortex; HP, hippocampus; S1, primary somatosensory cortex area; SMA, supplementary motor area*.

**Table 4:** Data of bivariate GCA of placebo vs. nocebo of brain GCA of fit signed-path coefficients.

		MNI				
BA	R/L	*X*	*Y*	*Z*	Voxel	Z-score
ACC to whole brain						
VMPFC	L	−12	45	−18	183	4.697
PHP	L	−33	−15	−15	191	6.2265
THS	R	6	0	−3	375	6.1257
CAU	L	−15	6	15	157	4.5322
S1	L	−6	−42	57	126	−5.3669
SMA	R	6	−3	75	131	−3.7084
Whole brain to ACC						
IC	R	30	−6	18	932	6.2055
DLPFC	L	−39	57	−12	277	−6.2768
IC	L	−24	15	12	332	−6.2703
PCC	L	−9	−39	33	209	−5.6811

*BA, Brodmann areas; MNI, Montreal Neurological Institute. VMPFC, ventromedial prefrontal cortex; PHP, parahippocampal gyrus; THS, thalamus; CAU, caudate; S1, primary somatosensory cortex area; SMA, supplementary motor area; IC, insular cortex; DLPFC, dorsolateral prefrontal cortex; PCC, posterior cingulate cortex*.

### Multivariate GCA

**Figure 7:**
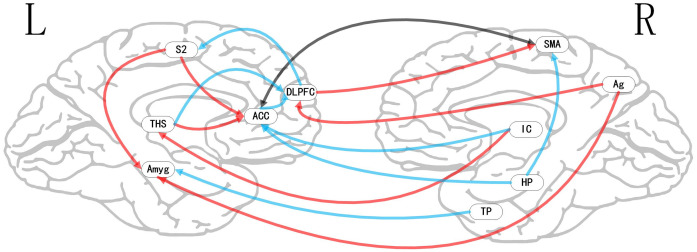
Map illustrating brain of two status (placebo vs. Pain) of multivariate GCA signed-path coefficients. Some brain regions only showed projection position. Blue/red arrows designate remarkably greater repression/activation of ensuing target activity in placebo vs. pain. Black arrow designates the bidirectional adjustment process between brain areas. ACC, anterior cingulate cortex; AMYG, amygdala; THS, thalamus; AG, angular gyrus; TP, temporal pole; HP, hippocampus; DLPFC, dorsolateral prefrontal cortex; IC, insular cortex; S2, secondary somatosensory cortex area; SMA, supplementary motor area.

**Figure 8:**
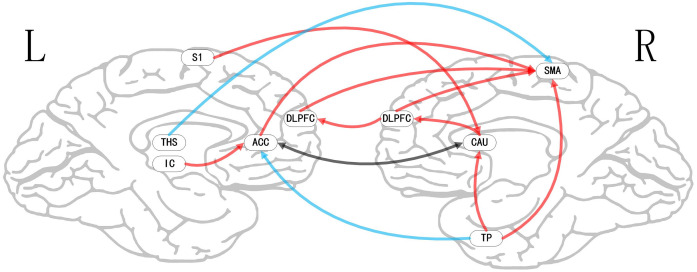
Map illustrating brain map of two-status (nocebo vs. pain) of multivariate GCA signed-path coefficients. Some brain regions only showed projection position. Blue/red arrows show remarkably greater repression/activation of ensuing target activity in nocebo vs. pain. The black arrow shows a bidirectional adjustment process between brain areas. ACC, anterior cingulate cortex; TP, temporal pole; THS, thalamus; CAU, caudate; DLPFC, dorsolateral prefrontal cortex; IC, insular cortex; HP, hippocampus; S1, primary somatosensory cortex area; SMA, supplementary motor area.

**Figure 9:**
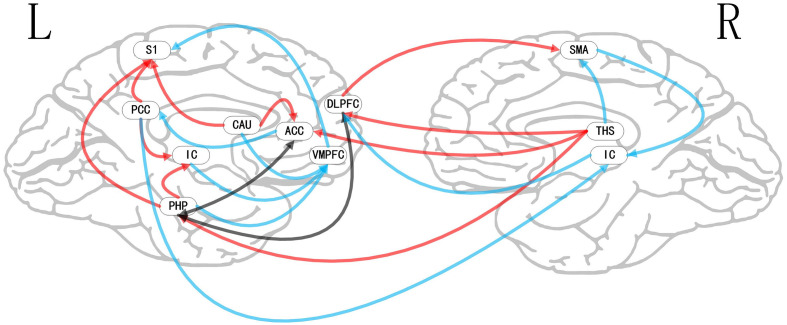
Map illustrating brain map of two-status (placebo vs. nocebo) of multivariate GCA signed-path coefficients. Some brain regions only showed projection position. Blue/red arrows show remarkably greater repression/activation of ensuing target activity in placebo vs. nocebo. The black arrow shows a bidirectional adjustment process between brain areas. ACC, anterior cingulate cortex; VMPFC, ventromedial prefrontal cortex; PHP, parahippocampal gyrus; THS, thalamus; CAU, caudate; S1, primary somatosensory cortex area; SMA, supplementary motor area; IC, insular cortex; DLPFC, dorsolateral prefrontal cortex; PCC, posterior cingulate cortex.

i. In the placebo condition, multivariate GCA integrating regions determined in bivariate GC assessments exhibited unique results not seen in bivariate analysis. (1) IC activity forecasted ensuing deactivation of the ACC; ACC activity forecasted ensuing deactivation of the DLPFC; DLPFC activity forecasted ensuing deactivation of the S2. (2) Activity in the HP forecasted ensuing ACC and SMA deactivation. Activity in the SMA estimated ensuing ACC deactivation. Activity in the IC forecasted ensuing induction of the THS and ACC deactivation. (3) Activity in the AG reflected ensuing stimulation of the DLPFC; activity in the DLPFC forecasted ensuing SMA activation. (4) Activity in the IC reflected ensuing THS activation; activity in the THS forecasted ensuing ACC activation; activity in the ACC reflected ensuing SMA activation (Tables s 5–7, Figure 7).
ii. In nocebo conditions, multivariate GCA integrating regions determined in the bivariate GC analyses returned unique data not seen in bivariate assessment. (1) Activity in the TP reflected ensuing ACC deactivation. (2) Activity in the CAU forecasted ensuing ACC deactivation. (3) Activity in the THS reflected ensuing SMA deactivation. (4) Activity in the IC forecasted ensuing ACC activation; activity in the ACC reflected ensuing SMA activation. (5) Activity in the S1 forecasted ensuing CAU activation; activity in the CAU forecasted ensuing right DLPFC activation; activity in the right DLPFC forecasted ensuing SMA activation (Tables s 8–10, Figure 8).
iii. In differences between placebo and nocebo conditions, multivariate GCA integrating regions determined in bivariate GC assessments exhibited unique results not seen in bivariate analysis. (1) ACC activity forecasted ensuing deactivation of the PHP and PCC; VMPFC activity forecasted ensuing deactivation of the S1; PHP activity forecasted ensuing deactivation of the VMPFC, and forecasted ensuing activation of the ACC, S1, DLPFC, and left IC; THS activity forecasted ensuing deactivation of the SMA, and forecasted ensuing activation of the ACC, PHP and DLPFC; CAU activity forecasted ensuing deactivation of the VMPFC, and forecasted ensuing activation of the ACC and S1; SMA activity forecasted ensuing deactivation of the right IC; IC activity forecasted ensuing deactivation of the DLPFC and PCC, and forecasted ensuing activation of the SMA; DLPFC activity forecasted ensuing activation of the PHP and SMA; left IC activity forecasted ensuing deactivation of the VMPFC; PCC activity forecasted ensuing activation of the S1 and left IC; SMA activity forecasted ensuing deactivation of the CAU, forecasted ensuing activation of the THS (Tables s 11–13, Figure 9).

**Table 5:** Multivariate GCA result of two-status (placebo vs. Pain) comparison fit signed-path coefficients.

	ACC	AMYG	THS	AG	TP	HP	DL	IC	S2	SMA
ACC	0.0064	−0.0577	−0.0623	0.0651	0.0492	0.0297	**−0.0743**	0.0260	−0.0127	**0.1015**
AMYG	0.1489	0.0086	−0.1189	−0.0471	0.0298	−0.0516	−0.0525	0.0034	−0.0187	−0.0384
THS	**0.0806**	0.0041	0.0083	−0.0417	−0.0107	0.0252	**−0.0465**	−0.0305	0.0681	0.0036
AG	−0.1056	**0.0747**	0.0196	−0.0030	−0.0300	−0.0445	**0.0675**	0.0587	−0.0420	0.0092
TP	−0.1623	**−0.0960**	−0.1259	−0.0310	−0.0348	−0.0364	0.0067	0.0517	−0.0034	−0.0185
HP	**−0.0825**	0.0346	0.0129	0.0258	0.0168	0.0325	−0.0127	0.0713	−0.0339	**−0.1075**
DL	0.1011	0.0043	−0.0433	0.0456	−0.0478	−0.0213	0.0212	0.0080	**−0.1541**	**−0.1419**
IC	**−0.0843**	0.0077	**0.0756**	−0.0265	−0.0029	0.0069	0.0369	−0.0342	0.0069	−0.0022
S2	**0.0679**	**0.0612**	−0.0482	0.0429	−0.0211	0.0041	−0.0029	−0.0684	−0.0196	0.0103
SMA	**−0.0737**	0.0417	0.0518	−0.0218	−0.0035	0.0304	0.0301	−0.0508	−0.0029	−0.0159

*Group mean path coefficients are shown with predictions going from row to column. Group means in bold are significantly different between placebo and pain, where a within-group effect was also observed in placebo status (Table 5; two-tailed tests; all *p* ≤ 0.05). ACC, anterior cingulate cortex; AMYG, amygdala; THS, thalamus; AG, angular gyrus; TP, temporal pole; HP, hippocampus; DLPFC, dorsolateral prefrontal cortex; IC, insular cortex; S2, secondary somatosensory cortex area; SMA, supplementary motor area*.

**Table 6:** GCA multivariate data of placebo status of coefficients of fit signed path.

	ACC	AMYG	THS	AG	TP	HP	DL	IC	S2	SMA
ACC	**0.7528**	−0.0136	0.0045	0.0492	0.0048	0.0145	**−0.0381**	0.0422	**−0.0387**	**0.0598**
AMYG	0.0279	**0.7862**	−0.0443	**−0.0364**	**0.0446**	**−0.0567**	−0.0207	0.0005	−0.0303	−0.0245
THS	**0.0406**	−0.0076	**0.7687**	−0.0150	0.0043	0.0267	**−0.0408**	**−0.0430**	0.0185	0.0093
AG	−0.0213	**0.0783**	−0.0071	**0.7867**	−0.0371	−0.0210	**0.0653**	0.0266	−0.0382	0.0151
TP	−0.0485	**−0.0505**	−0.0572	−0.0205	**0.7789**	−0.0495	0.0239	−0.0147	0.0134	**−0.0566**
HP	**−0.0930**	**0.0463**	0.0018	0.0093	0.0159	**0.7896**	0.0337	**0.1242**	0.0322	**−0.0886**
DL	0.0709	0.0200	0.0062	0.0093	−0.0483	−0.0062	**0.7921**	−0.0670	**−0.1161**	**0.1255**
IC	**−0.0463**	**0.0255**	**0.0606**	−0.0037	−0.0043	**−0.0254**	0.0286	**0.7746**	0.0210	−0.0047
S2	**0.0312**	**0.0500**	**−0.0706**	**0.0313**	0.0074	−0.0181	−0.0024	**−0.0626**	**0.7870**	0.0349
SMA	**−0.0576**	0.0223	0.0228	**−0.0386**	0.0093	**0.0406**	0.0171	−0.0117	0.0144	**0.7715**

*Group mean path coefficients with predictions going from row to column are shown. Group means in bold are significantly different from zero (two-tailed tests; all *p* ≤ 0.05). ACC, anterior cingulate cortex; AMYG, amygdala; THS, thalamus; AG, angular gyrus; TP, temporal pole; HP, hippocampus; DLPFC, dorsolateral prefrontal cortex; IC, insular cortex; S2, secondary somatosensory cortex area; SMA, supplementary motor area*.

**Table 7:** GCA multivariate data of pain comparison of coefficients of fit signed-path.

	ACC	AMYG	THS	AG	TP	HP	DL	IC	S2	SMA
ACC	**0.7465**	**0.0441**	**0.0669**	−0.0160	**−0.0444**	**−0.0153**	**0.0362**	0.0161	−0.0260	−0.0418
AMYG	**−0.1209**	**0.7776**	0.0747	0.0107	0.0148	−0.0051	**0.0318**	−0.0029	−0.0116	0.0139
THS	**−0.0400**	−0.0117	**0.7605**	0.0267	0.0150	0.0015	0.0057	−0.0125	**−0.0496**	0.0057
AG	**0.0842**	0.0036	−0.0267	**0.7896**	−0.0072	0.0235	−0.0022	−0.0321	0.0038	0.0059
TP	**0.1137**	**0.0454**	**0.0687**	0.0105	**0.8137**	−0.0131	0.0173	**−0.0664**	0.0169	−0.0381
HP	−0.0105	0.0117	−0.0112	−0.0165	−0.0010	**0.7571**	**0.0464**	0.0529	**0.0662**	0.0189
DL	−0.0302	0.0157	0.0495	−0.0363	−0.0004	0.0152	**0.7709**	−0.0750	0.0381	−0.0164
IC	**0.0380**	0.0178	−0.0150	**0.0228**	−0.0014	**−0.0324**	−0.0083	**0.8088**	0.0141	−0.0025
S2	−0.0366	−0.0112	−0.0224	−0.0116	**0.0285**	−0.0222	0.0004	0.0058	**0.8066**	0.0247
SMA	0.0161	−0.0195	−0.0290	−0.0169	0.0128	0.0101	−0.0131	**0.0391**	0.0173	**0.7874**

*Group mean path coefficients with predictions going from row to column are shown. Group means in bold are significantly different from zero (two-tailed tests; all *p* ≤ 0.05). ACC, anterior cingulate cortex; AMYG, amygdala; THS, thalamus; AG, angular gyrus; TP, temporal pole; HP, hippocampus; DLPFC, dorsolateral prefrontal cortex; IC, insular cortex; S2, secondary somatosensory cortex area; SMA, supplementary motor area*.

**Table 8:** GCA multivariate data of two-status (nocebo vs. pain) comparison fit signed-path coefficients.

	ACC	TP	THS	CAU	DL(L)	IC	HP	DL(R)	S1	SMA
ACC	0.0155	0.0932	−0.0596	**0.1102**	−0.0936	−0.0782	0.0406	−0.0978	0.0512	**0.3036**
TP	**−0.1064**	0.0276	−0.0588	**0.1009**	0.0022	−0.0498	−0.0047	−0.0360	−0.0132	**0.1333**
THS	0.0465	−0.0142	−0.0088	−0.0251	−0.0585	−0.0167	−0.0254	−0.0022	−0.0262	**−0.0860**
CAU	**−0.1340**	0.0291	0.0399	−0.0468	0.0909	−0.0038	0.0381	**0.1115**	0.0090	−0.0588
DL(L)	0.0217	−0.0237	−0.0121	0.0104	0.0200	−0.0044	−0.0148	0.0277	0.0096	**0.0540**
IC	**0.0485**	−0.0135	0.0015	−0.0112	0.0116	−0.0362	−0.0295	−0.0596	0.0129	−0.0655
HP	−0.0357	−0.0316	0.0605	0.0304	0.0796	0.0342	0.0108	−0.0383	−0.0085	0.0885
DL(R)	0.0452	−0.0284	−0.0197	0.0052	**−0.1611**	−0.0380	0.0362	0.0150	0.0469	**0.0869**
S1	−0.0562	−0.0078	−0.0303	**0.0726**	0.0183	−0.0412	0.0340	0.0000	−0.0238	−0.0460
SMA	−0.0106	−0.0169	0.0066	−0.0065	0.0041	0.0077	−0.0303	−0.0149	0.0258	0.0119

*Group mean path coefficients with predictions going from row to column, are shown. Group means in bold are significantly different between nocebo and pain, where a within-group effect was also present in nocebo status (Table 8; two-tailed tests; all *p* ≤ 0.05). ACC, anterior cingulate cortex; TP, temporal pole; THS, thalamus; CAU, caudate; DLPFC, dorsolateral prefrontal cortex; IC, insular cortex; HP, hippocampus; S1, primary somatosensory cortex area; SMA, supplementary motor area*.

**Table 9:** GCA multivariate data of nocebo status comparison of coefficients of fit signed path.

	ACC	TP	THS	CAU	DL(L)	IC	HP	DL(R)	S1	SMA
ACC	**0.7715**	0.0316	−0.0306	**0.0783**	−0.0056	−0.0135	**0.0499**	−0.0282	0.0481	**0.1851**
TP	**−0.0422**	**0.7997**	−0.0253	**0.0486**	**0.0650**	0.0125	0.0049	−0.0012	0.0011	**0.0693**
THS	0.0266	−0.0040	**0.7858**	−0.0266	−0.0496	0.0064	−0.0118	−0.0181	−0.0290	**−0.0505**
CAU	**−0.0754**	0.0250	0.0419	**0.7236**	0.0336	−0.0111	0.0050	**0.1148**	−0.0174	−0.0005
DL(L)	0.0168	**−0.0385**	**−0.0351**	−0.0093	**0.7664**	0.0103	0.0218	0.0265	0.0232	**0.0626**
IC	**0.0345**	**−0.0455**	−0.0232	−0.0110	**−0.0813**	**0.7914**	**−0.0472**	**−0.0678**	0.0481	−0.0129
HP	0.0141	**−0.0404**	0.0228	**0.0380**	0.0030	0.0682	0.8047	0.0033	−0.0291	0.0059
DL(R)	0.0131	−0.0215	**0.0432**	−0.0042	**−0.0672**	−0.0058	**0.0447**	**0.7804**	0.0232	**0.1203**
S1	−0.0209	0.0058	0.0346	**0.0431**	0.0292	**−0.0512**	0.0200	−0.0049	**0.7920**	−0.0134
SMA	−0.0026	−0.0171	0.0006	−0.0073	−0.0521	−0.0150	−0.0169	−0.0236	0.0135	**0.7857**

*Group mean path coefficients with predictions going from row to column are shown. Group means in bold are significantly different from zero (two-tailed tests; all *p* ≤ 0.05). ACC, anterior cingulate cortex; TP, temporal pole; THS, thalamus; CAU, caudate; DLPFC, dorsolateral prefrontal cortex; IC, insular cortex; HP, hippocampus; S1, primary somatosensory cortex area; SMA, supplementary motor area*.

**Table 10:** GCA multivariate data of pain comparison of coefficients of fit signed path.

	ACC	TP	THS	CAU	DL(L)	IC	HP	DL(R)	S1	SMA
ACC	**0.7560**	**−0.0616**	0.0289	−0.0319	**0.0880**	**0.0648**	0.0093	**0.0696**	−0.0031	**−0.1185**
TP	**0.0642**	**0.7721**	0.0335	**−0.0522**	0.0628	**0.0624**	0.0096	0.0348	0.0143	−0.0639
THS	−0.0199	0.0103	**0.7947**	−0.0016	0.0088	0.0230	0.0136	−0.0159	−0.0028	0.0355
CAU	**0.0586**	−0.0041	0.0020	**0.7704**	−0.0573	−0.0073	−0.0331	0.0033	−0.0264	**0.0583**
DL(L)	−0.0049	−0.0147	−0.0230	**−0.0197**	**0.7464**	0.0146	**0.0366**	−0.0012	0.0136	0.0086
IC	−0.0140	−0.0321	−0.0247	0.0002	**−0.0929**	**0.8276**	−0.0176	−0.0083	0.0351	0.0526
HP	**0.0498**	−0.0088	−0.0377	0.0076	−0.0766	**0.0340**	**0.7939**	0.0416	−0.0206	**−0.0826**
DL(R)	**−0.0321**	0.0069	**0.0628**	−0.0093	**0.0939**	0.0322	0.0085	**0.7654**	−0.0237	0.0334
S1	**0.0352**	0.0136	**0.0649**	−0.0295	0.0109	−0.0100	−0.0140	−0.0049	**0.8158**	0.0326
SMA	0.0079	−0.0002	−0.0060	−0.0008	**−0.0563**	**−0.0227**	0.0134	−0.0087	−0.0122	**0.7738**

*Group mean path coefficients with predictions going from row to column are shown. Group means in bold are significantly different from zero (two-tailed tests; all *p* ≤ 0.05). ACC, anterior cingulate cortex; TP, temporal pole; THS, thalamus; CAU, caudate; DLPFC, dorsolateral prefrontal cortex; IC, insular cortex; HP, hippocampus; S1, primary somatosensory cortex area; SMA, supplementary motor area*.

**Table 11:** GCA multivariate data of two-status (placebo vs. nocebo) comparison fit signed-path coefficients.

	ACC	VMPFC	PHP	THS	CAU	S1	SMA	IC(r)	DL	IC(l)	PCC
ACC	−0.0034	−0.0280	**−0.0830**	−0.0201	−0.0629	0.0440	0.0402	0.0073	−0.0194	−0.0461	**−0.0757**
VMPFC	0.0776	−0.0181	0.0455	−0.0519	−0.0538	**−0.1430**	0.0361	0.0084	−0.0294	−0.0908	0.0270
PHP	**0.1467**	**−0.0854**	−0.0089	−0.0038	−0.0526	**0.1490**	−0.0393	0.0663	**0.0579**	**0.1132**	0.0269
THS	**0.1480**	0.0330	**0.1045**	0.0117	−0.0296	−0.0003	**−0.0410**	−0.0491	**0.0573**	0.1164	−0.0071
CAU	**0.0798**	**−0.0633**	0.0240	−0.0073	−0.0283	**0.0625**	−0.0137	0.0675	0.0003	0.0741	−0.0134
S1	−0.0414	0.0400	−0.0248	−0.0427	−0.0472	−0.0372	−0.0062	−0.0151	0.0151	0.0254	−0.0092
SMA	−0.0927	−0.0222	0.0290	−0.0089	−0.0588	0.0046	0.0000	**−0.1787**	−0.0298	−0.0781	0.0956
IC(r)	−0.0133	−0.0116	−0.0328	0.0156	−0.0434	−0.0327	−0.0143	0.0033	**−0.0251**	0.0420	**−0.0487**
DL	0.0685	0.0056	**0.1024**	0.0174	−0.0624	0.0384	**0.0756**	−0.0828	−0.0066	−0.0633	0.0710
IC(l)	0.0391	**−0.0516**	0.0027	0.0038	0.0161	0.0202	0.0156	−0.0430	0.0076	0.0356	−0.0219
PCC	0.0272	−0.0165	−0.0075	−0.0134	−0.0383	**0.0946**	−0.0026	0.0276	0.0128	**0.1057**	0.0049

*Group mean path coefficients with predictions going from row to column are shown. Group means in bold are significantly different between placebo and nocebo, where a within-group effect was also present in placebo/nocebo status (Tables s 12, 13; two-tailed tests; all *p* ≤ 0.05). ACC, anterior cingulate cortex; VMPFC, ventromedial prefrontal cortex; PHP, parahippocampal gyrus; THS, thalamus; CAU, caudate; S1, primary somatosensory cortex area; SMA, supplementary motor area; IC, insular cortex; DLPFC, dorsolateral prefrontal cortex; PCC, posterior cingulate cortex*.

**Table 12:** GCA multivariate data of placebo status of coefficients of fit signed path.

	ACC	VMPFC	PHP	THS	CAU	S1	SMA	IC	DL	IC	PCC
ACC	**0.7652**	0.0036	−0.0102	0.0091	−0.0350	−0.0047	**0.0398**	0.0159	−0.0071	**−0.0793**	−0.0112
VMPFC	0.0287	**0.7737**	**0.0625**	**−0.0517**	−0.0298	**−0.0748**	0.0299	−0.0480	−0.0286	**−0.1570**	**0.0416**
PHP	**0.0746**	**−0.0656**	**0.7762**	0.0099	−0.0183	**0.0993**	0.0097	**0.0994**	**0.0319**	−0.0014	0.0327
THS	−0.0036	**0.0678**	0.0103	**0.7474**	−0.0379	**0.0958**	−0.0094	−0.0505	0.0116	0.0317	0.0395
CAU	**0.0431**	**−0.0394**	**0.0331**	0.0015	**0.7681**	**0.0545**	**−0.0214**	0.0295	−0.0066	0.0485	−0.0175
S1	−0.0202	0.0233	−0.0220	**−0.0399**	−0.0167	**0.7553**	−0.0024	−0.0417	0.0215	−0.0491	−0.0069
SMA	−0.0668	−0.0003	0.0127	−0.0022	−0.0137	−0.0239	**0.7536**	−0.0222	0.0123	−0.0461	0.0178
IC	**−0.0422**	0.0081	**−0.0273**	0.0059	−0.0352	−0.0015	**−0.0398**	**0.7579**	0.0121	**0.1201**	−0.0105
DL	0.0199	0.0483	**0.0842**	0.0001	−0.0151	0.0010	−0.0271	**−0.1308**	**0.7815**	**−0.1895**	**0.0649**
IC	0.0147	**−0.0414**	**0.0224**	−0.0023	**−0.0242**	**0.0399**	0.0117	**−0.0310**	**0.0192**	**0.7844**	−0.0029
PCC	−0.0158	0.0009	**−0.0447**	−0.0014	0.0032	0.0146	−0.0038	−0.0127	−0.0055	0.0107	**0.7725**

*Group mean path coefficients with predictions going from row to column are shown. Group means in bold are significantly different from zero (two-tailed tests; all *p* ≤ 0.05). ACC, anterior cingulate cortex; VMPFC, ventromedial prefrontal cortex; PHP, parahippocampal gyrus; THS, thalamus; CAU, caudate; S1, primary somatosensory cortex area; SMA, supplementary motor area; IC, insular cortex; DLPFC, dorsolateral prefrontal cortex; PCC, posterior cingulate cortex*.

**Table 13:** GCA multivariate data of nocebo status comparison of coefficients of fit signed path.

nocebo	ACC	VMPFC	PHP	THS	CAU	S1	SMA	IC	DL	IC	PCC
ACC	**0.7687**	0.0316	**0.0728**	0.0292	0.0279	−0.0487	−0.0004	0.0087	0.0123	−0.0332	**0.0645**
VMPFC	**−0.0490**	**0.7919**	0.0170	0.0002	0.0240	0.0683	−0.0063	−0.0564	0.0008	−0.0662	0.0146
PHP	**−0.0720**	0.0198	**0.7851**	0.0137	0.0343	−0.0497	0.0490	0.0330	−0.0260	**−0.1146**	0.0058
THS	**−0.1516**	0.0348	**−0.0942**	**0.7356**	−0.0083	**0.0961**	**0.0316**	−0.0013	**−0.0458**	−0.0847	0.0467
CAU	−0.0367	0.0239	0.0091	0.0089	**0.7965**	−0.0079	−0.0077	−0.0380	−0.0068	−0.0256	−0.0040
S1	0.0212	−0.0167	0.0029	0.0028	0.0305	**0.7925**	0.0038	−0.0265	0.0064	**−0.0745**	0.0023
SMA	0.0259	0.0219	−0.0163	0.0067	0.0451	−0.0285	**0.7537**	**0.1564**	**0.0421**	0.0319	**−0.0778**
IC	−0.0289	0.0197	0.0055	−0.0098	0.0081	0.0312	**−0.0255**	**0.7545**	**0.0372**	**0.0782**	**0.0383**
DL	−0.0485	0.0427	−0.0182	−0.0173	0.0473	−0.0375	**−0.1026**	−0.0479	**0.7882**	**−0.1262**	−0.0061
IC	−0.0245	0.0102	0.0197	−0.0061	**−0.0403**	0.0198	−0.0039	0.0120	0.0116	**0.7488**	0.0190
PCC	−0.0430	0.0174	−0.0372	0.0120	0.0416	**−0.0799**	−0.0013	−0.0403	−0.0183	**−0.0949**	**0.7676**

*Group mean path coefficients with predictions going from row to column are shown. Group means in bold are significantly different from zero (two-tailed tests; all *p* ≤ 0.05). ACC, anterior cingulate cortex; VMPFC, ventromedial prefrontal cortex; PHP, parahippocampal gyrus; THS, thalamus; CAU, caudate; S1, primary somatosensory cortex area; SMA, supplementary motor area; IC, insular cortex; DLPFC, dorsolateral prefrontal cortex; PCC, posterior cingulate cortex*.

## Discussion

Herein, we established that the application of an analgesic patch with pain relief expectation triggered a remarkable VAS reduction. On the contrary, giving an algetic patch with the expectation of pain enhancement evoked a remarkable VAS increase. We also found that fMRI signals are altered between placebo/nocebo status and pain status.

### Common Characteristics of Placebo and Nocebo Responses

Brain regions with activity in pain networks are defined as pain-related networks (Apkarian et al., 2005; Tracey, 2008). Some pain-related networks are crucial in the generation along with the transmission of feeling. For example, IC and ACC participate in emotional component. ACC lesions are known to abolish the affective components of pain, without affecting location of pain stimulation. The IC is also involved in emotional components owing to its extensive connections to ACC (Vogt et al., 1996; Ingvar, 1999). Here, we found a negative lagged-temporal correlation between IC and ACC and between ACC and S2 in placebo response, which may lead to decreased activation of brain areas. It suggests that the pain linked network diminishes transmission along with the processing of pain information under placebo analgesia, and reduces the sensitivity of every brain part in the matrix to sensory information. In nocebo response, we found multiple positive lagged-temporal correlations in pain-related brain areas, including ACC, IC, SMA, CAU, DLPFC. Unlike placebo response, the brain escalates transmission coupled with analysis of pain information in the nocebo response, causing more pain sensations.

As a sensory transmission area, the IC perceives information like pain, itching, as well as sensual touch from the THS ventromedial nucleus, before passing it to the ACC for processing of sensory information (Craig et al., 2000). The negative lagged-temporal correlation between IC and ACC indicates that placebo response is likely to reduce the transfer of sensory information by the IC, and also reduces the ACC’s speed of processing speed sensory information, causing analgesic effects. But in the nocebo response, the lagged-temporal correlation was reversed. It suggests that the above network regulation may also be reversed. The above networks may play a role in hyperalgesia by improving the ACC’s processing speed of sensory information.

In the placebo response, we found a negative lagged-temporal correlation between ACC and DLPFC. While in the nocebo response, this relationship was reversed. The DLPFC has an indispensable role in emotion control along with pain regulation (Fields, 2000; Kong et al., 2013). This illustrates that this relationship affects the function of DLPFC in pain-related networks in placebo along with nocebo responses. In addition to the above functions, the DLPFC is also an important part of descending pain modulatory system, which negatively regulates IC and PAG activation (Eippert et al., 2009). The differences in the above lagged-temporal correlations illustrate that both placebo along with nocebo responses may be involved in the activation changes of the descending pain modulatory system. Related brain networks have a role in placebo analgesia, as well as nocebo hyperalgesia by inhibiting or activating this system.

The reward system constitutes a cluster of neural structures that account for incentive salience, associative learning, coupled with positive emotions, especially those involving pleasure as a core component (Berridge and Kringelbach, 2015; Schultz, 2015). This system consists of the ventral striatum, PFC, ACC, IC, THS, along with AMYG (Richard et al., 2013; Berridge and Kringelbach, 2015). We found multiple positive lagged-temporal correlations in placebo response involving IC, THS, ACC, and SMA. This illustrates that the activation of the reward system may be altered with a placebo intervention. Stimulation of the reward system elevates the levels of neurotransmitters, including dopamine and GABA (gamma-aminobutyric acid; Yager et al., 2015), which may promote “happiness” and suppress pain sensation. The mechanisms involved in the reward system are controversial. Scott et al. (2007) opined that placebo response cause dopamine release and that dopamine is associated with placebo response. However, Wrobel et al. (2014) opined that dopamine may not causally participate in placebo response but might be related to reward processing and learning of placebo response. Our results suggest that the placebo response may activate the reward system and may lead to the release of dopamine. In the nocebo response, our data show that the THS can predict SMA deactivation. Reduced thalamus activity may affect dopamine secretion, which is the exact opposite of the placebo response.

### Individual Characteristics of Placebo and Nocebo Responses

The HP is associated with approach-avoidance conflict that occurs when a potentially rewarding or punishing situation is presented. Thus, the ensuing decision-making is anxiety associated (O’Neil et al., 2015). Anxiety is regarded as a remarkable cause of nocebo response, which may suppress placebo response (Thibodeau et al., 2013; Woo, 2015). The nervous center that causes anxiety is linked to the ACC, AMYG, HP, along with brainstem. Negative HP modulation may decrease the incidence of anxiety, while ACC overactivation causes anxiety (Lieberman and Eisenberger, 2009; Zeidan et al., 2014). We found a negative lagged-temporal correlation between HP and ACC in the placebo response, but not in the nocebo response. The negative lagged-temporal correlation may inhibit anxiety and reduce its occurrence, which may influence placebo response.

IC, THS, ACC, and SMA are associated with the OMAS (Colloca and Benedetti, 2016), we found multiple positive lagged-temporal correlations in the above brain areas. Scott et al. (2008) tasked subjects to go through a 20-min pain challenge, then observed placebo-elevated opioid neurotransmission in the ACC, orbitofrontal and IC, nucleus accumbens, AMYG, as well as periaqueductal gray, along with dopamine activation (DA) in the ventral basal ganglia. The multiple positive lagged-temporal correlations may activate the OMAS, which would produce more opioids. Additionally, opioids have analgesic effects and are associated with pleasure and euphoria (Maltoni, 2008). Although the brainstem is also implicated in OMAS activation (Petrovic et al., 2002; Grahl et al., 2018), no lagged-temporal correlation between the brainstem and other brain areas was found in our results. This suggests that ACC and brainstem may not have a direct temporal correlation.

The DLPFC, ACC, THS, and AMYG are part of the emotion pathway (Stevens et al., 2011). The AMYG is a pivotal node of the emotional cascade, receiving emotional signals from other brain areas in the emotional pathway (Amunts et al., 2005; Shi et al., 2019). The THS, particularly the hypothalamus, constitutes the earliest described brain structure closely linked to emotion. In emotion conduction, the THS has active nerve fiber connections with numerous brain portions. For example, the medial dorsal nucleus of THS is linked to the PFC area (Cross et al., 2013; Shi et al., 2019). Furthermore, the anterior nucleus of THS is linked to mammillary bodies *via* the fornix, which in turn connects it to the HP, as well as the cingulate cortex (CC; Aggleton et al., 2014; Shi et al., 2019). We found multiple positive lagged-temporal correlations in the placebo response, including THS, ACC, and AMYG. The relationships may accelerate the transmission and analysis of emotions, generating more positive emotions. This phenomenon is the opposite of the brain’s response to negative emotions (depression and anxiety), indicating the brain positive response to a placebo.

Here, we found that TP and CAU can predict the ACC deactivation in nocebo response. The temporal lobe plays an indispensable role in emotional processes, as well as recognizes familiar facial expressions and comprehending a person’s emotions when different from body language. TP is also thought to have a role in emotional empathy precipitation along with emotional stability enhancement (Olson et al., 2007; Shi et al., 2019). The negative lagged-temporal correlation between TP and ACC may affect the functions of emotional stability involved in TP and thus affect emotional control in nocebo response. The CAU is part of the dorsal striatum and is implicated in responses to visual beauty and is associated with love generation (Aron et al., 2005; Ishizu and Zeki, 2011). The negative lagged-temporal correlation between CAU and ACC may impair judgment, thus aggravating the “gray life” in nocebo response.

### Limitation

While this work clearly shows the dynamic network of placebo analgesia along with nocebo hyperalgesia, two remarkable limitations are noted. First, because of a limited number of subjects, we could not conduct in-depth stratified participant analyses, including by gender or personalities. Second, the use of fMRI alone is too monotonous and the combination of task-fMRI and event-linked fMRI would have enriched the results. Thirdly, the sample size estimation in this study was based on previous literature, and the reliability of the results can be better improved by using the power analysis for sample size estimation.

## Conclusion

The results of this study suggest that both pain-related network and reward system are involved in placebo and nocebo responses. The placebo response mainly works by activating the reward system and inhibiting the pain-related network, while the nocebo response is the opposite. The placebo network also involves the activation of OMAS and emotion pathway, while the nocebo network involves the deactivation of emotional control. At the same time, through the construction of the GC network, we verified our hypothesis that placebo along with nocebo networks share some of the same brain areas, but the two networks also have their own unique structural features.

## Data Availability Statement

The raw data supporting the conclusions of this article will be made available by the authors, without undue reservation.

## Ethics Statement

The studies involving human participants were reviewed and approved by Ethics Committee of Zhujiang Hospital. The patients/participants provided their written informed consent to participate in this study.

## Author Contributions

YS, SC, and WW: study concept and design. YS, SC, and SH: acquisition, analysis, or interpretation of data. YS, YZ, and WW: drafting of the manuscript. JY, SH, and WW: critical revision of the manuscript. YS, YZ, GC, and SH: statistical analysis. JY: MRI technical support. WW and JY: study supervision.

## Conflict of Interest

The authors declare that the research was conducted in the absence of any commercial or financial relationships that could be construed as a potential conflict of interest.

## Publisher’s Note

All claims expressed in this article are solely those of the authors and do not necessarily represent those of their affiliated organizations, or those of the publisher, the editors and the reviewers. Any product that may be evaluated in this article, or claim that may be made by its manufacturer, is not guaranteed or endorsed by the publisher.

## Footnotes

^1^ http://www.restfmri.net

^2^ http://restfmri.net/forum/rest
